# Chromatographic separation of silver-111 from neutron-irradiated palladium target: toward direct labeling of radiotracers

**DOI:** 10.1186/s41181-023-00232-0

**Published:** 2023-12-20

**Authors:** Marianna Tosato, Andrea Gandini, Steffen Happel, Marine Bas, Antonietta Donzella, Aldo Zenoni, Andrea Salvini, Alberto Andrighetto, Valerio Di Marco, Mattia Asti

**Affiliations:** 1 Radiopharmaceutical Chemistry Section, Nuclear Medicine Unit, AUSL di Reggio Emilia: Azienda Unità Sanitaria Locale - IRCCS Tecnologie Avanzate e Modelli Assistenziali in Oncologia di Reggio Emilia, Via Amendola 2, 42122 Reggio Emilia, Italy; 2 Department of Chemical Sciences, University of Padova, 35131 Padua, Italy; 3 Laboratory of Applied Nuclear Energy, 27100 Pavia, Italy; 4 TrisKem International SAS, 35170 Brittany, France; 5 Department of Mechanical and Industrial Engineering, University of Brescia, 25123 Brescia, Italy; 6 Italian Institute of Nuclear Physics, Pavia Section, 27100 Pavia, Italy; 7 Italian Institute of Nuclear Physics, Legnaro National Laboratories, 35020 Legnaro, (Padova) Italy

**Keywords:** Silver-111, Theranostic pair, Theranostic, Radionuclide production, Radiochemical purification, Nuclear reactor, Palladium-110

## Abstract

**Background:**

Silver-111 is a promising *β*^−^-emitting radioisotope with ideal characteristics for targeted radionuclide therapy and associated single photon emission tomography imaging. Its decay properties closely resemble the clinically established lutetium-177, making it an attractive candidate for therapeutic applications. In addition, the clinical value of silver-111 is further enhanced by the existence of the positron-emitting counterpart silver-103, thus imparting a truly theranostic potential to this element. A so-fitting matching pair could potentially overcome the current limitations associated with the forced use of chemically different isotopes as imaging surrogates of lutetium-177, leading to more accurate and efficient diagnosis and treatment. However, the use of silver-111-based radiopharmaceuticals in vivo has faced obstacles due to the challenges related to its production and radiochemical separation from the target material. To address these issues, this study aims to implement a chromatographic separation methodology for the purification of reactor-produced silver-111. The ultimate goal is to achieve a ready-to-use formulation for the direct radiolabeling of tumour-seeking biomolecules.

**Results:**

A two-step sequence chromatographic process was validated for cold Ag-Pd separation and then translated to the radioactive counterpart. Silver-111 was produced *via* the ^110^Pd(n,γ)^111^Pd nuclear reaction on a natural palladium target and the subsequent *β*^−^-decay of palladium-111. Silver-111 was chemically separated from the metallic target *via* the implemented chromatographic process by using commercially available LN and TK200 resins. The effectiveness of the separations was assessed by inductively coupled plasma optical emission spectroscopy and γ-spectrometry, respectively, and the Ag^+^ retrieval was afforded in pure water. Recovery of silver-111 was > 90% with a radionuclidic purity > 99% and a separation factor of around 4.21·10^−4^.

**Conclusions:**

The developed separation method was suitable to obtain silver-111 with high molar activity in a ready-to-use water-based formulation that can be directly employed for the labeling of radiotracers. By successfully establishing a robust and efficient production and purification method for silver-111, this research paves the way for its wider application in targeted radionuclide therapy and precision imaging.

## Background

In recent years, the search and exploration of novel radioisotopes suitable for cancer therapy and diagnosis has blossomed. This remarkable growth reflects the exciting advancements and profound commitment of researchers in the field, propelling us toward a new era of cutting-edge medical procedures. Among the promising unconventional radionuclides, silver has newly attracted great attention as it possesses a plethora of radioisotopes (i.e. silver-103, silver-104 m/g and silver-111) with half-lives (*t*_1/2_) ranging from minutes to a few days and covering almost all the medically useful decay modes with the only exception of the α emission (Tosato and Asti 2023).

**Fig. 1:**
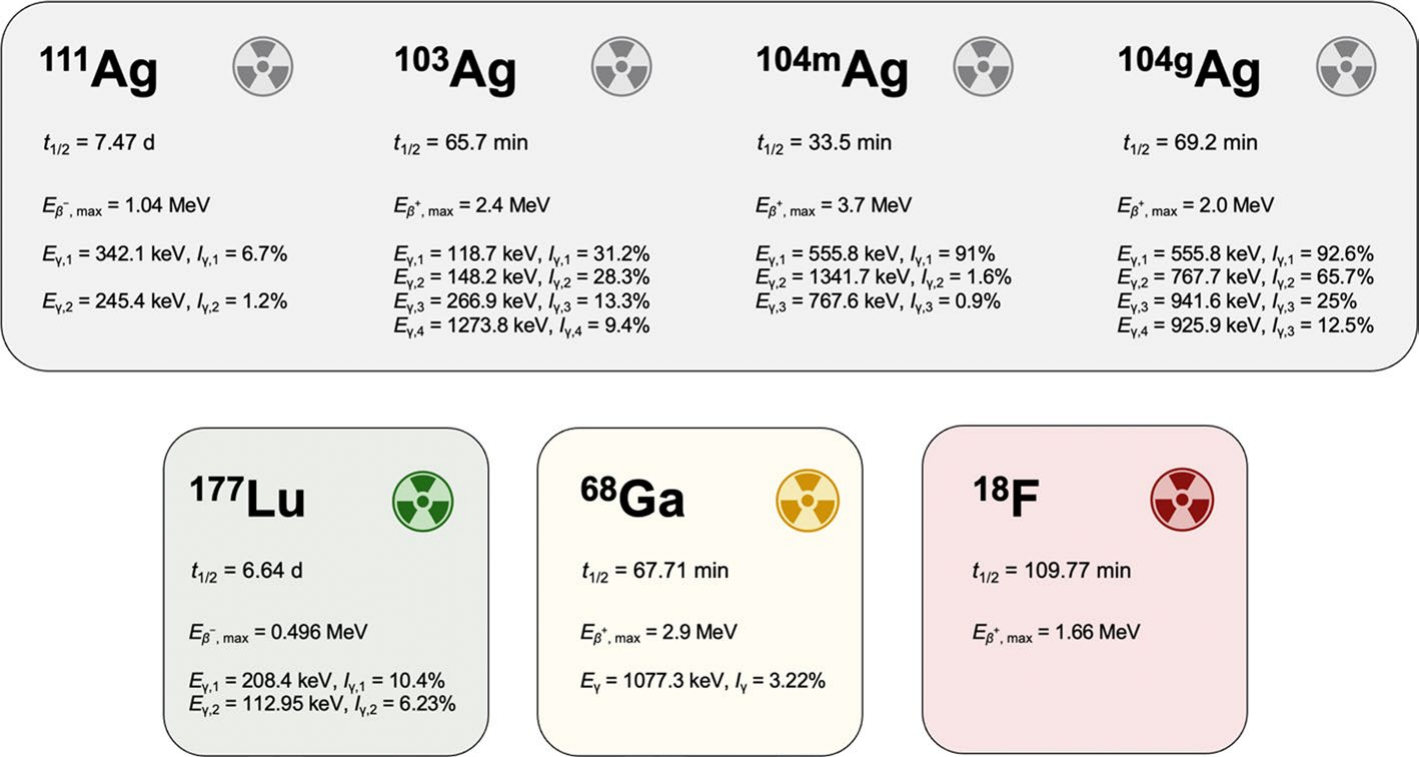
Comparison of the decay properties of silver radioisotopes (silver-111, silver-103, silver-104 m and silver-104 g), lutetium-177, gallium-68 and fluorine-18

In particular, the dual *β*^−^- and γ-emitter silver-111 (*t*_1/2_ = 7.47 d, *E*_*β*_^−^_, max_ = 1.04 MeV; *E*_γ,1_ = 342.1 keV, *I*_γ,1_ = 6.7%; *E*_γ,2_ = 245.4 keV, *I*_γ,2_ = 1.2%) could be used both for cancer therapy of tumor masses and related metastases, and associated Single Photon Emission Computed Tomography (SPECT) imaging (Mastren et al. 2018). Silver-111 possesses decay characteristics close to the clinically established lutetium-177 (Herrero Álvarez et al. 2021; Kostelnik and Orvig 2019; Mikolajczak et al. 2019). However, the absence of a diagnostic counterpart for the latter forces the use of chemically different radionuclides as imaging surrogates (e.g., gallium-68, fluorine-18). This “improper” isotope match can introduce bias in the biodistribution of the radiopharmaceutical, thus questioning if any quantitative information predicted from the diagnosis (such as the dosimetric calculations) may represent the behavior of the therapeutic agent (McNeil et al. 2021). Contrarily, silver-111 could be paired with a triplet of Positron Emission Tomography (PET) diagnostic counterparts: silver-103 (*t*_1/2_ = 65.7 min), silver-104 m (*t*_1/2_ = 33.5 min) and silver-104 g (*t*_1/2_ = 69.2 min) (Tárkányi et al. 2016). Silver-103 appears the most suited for effectively matching silver-111 in a clinical setting enabling accurate planning of personalized therapeutic interventions and in vivo monitoring of the disease progression during the treatment. Conversely, silver-104 m and silver-104 g exhibit highly abundant γ-emissions with energy close to 511 keV that may produce spurious coincidences. These wrong outputs could likely generate blurred PET images and cause quantification errors. The decay characteristics of each silver radioisotope along with lutetium-177, gallium-68 and fluorine-18 for the sake of comparison, are summarized in Fig. 1.

Notwithstanding these attractive properties, the use of silver radioisotopes for nuclear medicine purposes has been hindered so far by the challenges related to their production and separation from the target material. Moreover, the substantial absence of proper chelators able to stably incorporate silver radioisotopes in molecular vectors in vivo is a significant drawback as well. As a result, apart from a few pioneering studies regarding the complexation (radio)chemistry of silver-111 (Gyr et al. 1997; Tosato et al. 2020, 2022, 2023), no other investigations involving the use of silver radioisotopes for targeted nuclide therapy or molecular imaging have been reported to date.

Though still aimed at only preclinical research, the supply chain of silver-111 is surely the most advanced among the silver radioisotopes and its refinement inherits particular attention. The most viable silver-111 production route is the neutron irradiation of palladium targets *via* the ^110^Pd(n,γ)^111^Pd nuclear reaction and the subsequent decay of the intermediate palladium-111 (*t*_1/2_ = 23.4 min) to silver-111 (Alberto et al. 1992; El-Azony et al. 2022; Morselli et al. 2023). In this case, the use of highly enriched palladium-110 substrate is mandatory to obtain the desired radionuclide in high molar activity for medical applications as, in natural palladium, several additional silver species (e.g., silver-107, silver-109) would be concurrently produced due to side reactions occurring on the other palladium isotopes (Tosato and Asti 2023). Alternative production methods comprise deuteron-induced reactions through the ^110^Pd(d,n)^111^Ag direct pathway (El-Azony et al. 2022; Haymond et al. 1950; Hermanne et al. 2004) or the ^110^Pd(d,p)^111^Pd → ^111^Ag indirect route *(*Ditrói et al. 2012; El-Azony et al. 2022), α-induced ^108^Pd(α,p)^111^Ag and ^110^Pd(α,p2n)^111^Ag reactions (Hermanne et al. 2005), or silver-111 recovery from proton irradiated thorium matrix (Mastren et al. 2018). However, the low reaction cross-sections make these production routes less favorable alternatives compared to the reactor-production, which presently stands as the most convenient strategy. Moreover, the unavoidable presence of large amount of the long-lived silver-110m contaminant (t_1/2_ = 249.8 d) combined with the multi-step purification processes needed to extract silver-111 from the thorium matrix, introduces additional drawbacks in this production method.

The prospect of producing silver-111 *via* Isotope Separation On-Line (ISOL) technique has also been demonstrated in the context of a multidisciplinary research project named ISOLPHARM (ISOL technique for radioPHARMaceuticals) (Andrighetto et al. 2019; Ballan et al. 2020), but the limited number of ISOL facilities worldwide currently thwarts the affordability of this production route.

Several methods for the extraction of silver-111 from neutron-irradiated palladium targets have been explored. These methods include ion exchange chromatography (Aweda et al. 2013; Bauer et al. 1997; Lyle and Maghzian 1968; Mansur et al. 1995; Ohya et al. 2020; Ooe et al. 2019; Taylor 1961; Vimalnath et al. 2007), alumina adsorption (Khalid et al. 2000), liquid/liquid extraction (Alberto et al. 1992; Lahiri et al. 1997a, b), precipitation (Blackadar et al. 2022; Collins et al. 2014; Haymond et al. 1950; Sicilio et al. 1956; Zimen 1949), co-crystallization (Micheev et al. 1964) and electrodeposition (Griess and Rogers 1952). Nonetheless, many of these procedures involve the co-addition of macroscopic quantity of stable silver carrier or other chemical species (e.g. mercurous chloride), decreasing the molar activity and chemical purity of the obtained silver-111 under acceptable thresholds and resulting not suitable for radiopharmaceutical applications. This is not the case with the chromatographic methods that, even allowing a proper palladium depletion, have the principal drawback of providing Ag(I) in a large volume of highly concentrated acidic solutions that are unsuitable for the direct labelling of biological tumor-seeking vectors. This formulation forces evaporation steps and reconstruction in weakly acidic solutions, with the drawback of reducing the yields and possibly introducing stable contaminants into the final solution (Gracheva et al. 2019; McNeil et al. 2021).

Aiming to propel the implementation of silver-111 in both preclinical and future clinical contexts, this work intends to surmount the current limitation by introducing an innovative chromatographic separation process able to extract silver-111 selectively and efficiently from bulk palladium matrix and obtain it in a formulation suitable for the direct labelling of biological vectors.

## Methods

### Materials and methods

All chemicals were of trace metal grade and were used without further purification. Ultrapure nitric acid (69% *w*/*w*, Aristar for trace analysis) and hydrochloric acid (37%, Sigma Aldrich) were purchased from commercial suppliers. Ultrapure water was prepared on-site with a Millipore or Purelab Chorus water purification system (18 MΩ/cm). The main chemical separation was performed with di(2-ethylhexyl)orthophosphoric acid impregnated onto an inert support (LN resin, SA-LN-B01-S, particle size 50–100 μm, TrisKem International). Cartridges based on trioctylphosphine oxide (TK200 resin, 2 mL, particle size 50–100 μm, TrisKem International) served for refining the separation and for silver-111 concentration. Silver (1000 ± 2 mg/L, 2% *w*/*w* HNO_3_) and palladium standard solutions (1.000 ± 0.003 mg/L, 2% *w*/*w* HNO_3_) were purchased from Sigma Aldrich. Inductively Coupled Plasma Optical Emission Spectroscopy (ICP-OES) was performed to assess the effectiveness of the “cold” separation using an iCAP 7400 DUO Thermo Scientific instrument. Argon was used as an internal standard to monitor and correct the instrumental drift during the runs. The calibration solutions for ICP-OES were prepared by gravimetric serial dilution from element standard solutions at six different concentrations. Blank solutions were monitored regularly to ensure no Ag(I) nor Pd(II) cross-contamination during the measurements. Palladium stocks for irradiation purposes (purity 99.95%) were purchased from Goodfellow Cambridge Limited (UK). Gamma-ray spectrometry was employed to test the effective separation of silver-111 from the irradiated target material and was conducted using a Canberra GX2019 high-purity germanium detector, with a coaxial geometry one open-end closed-end facing window. For the instrument energy and efficiency calibrations a LEA multi-gamma source was used to acquire spectra at different heights from the detector.

### Silver and palladium weight distribution ratios

To derive the best separation conditions, the adsorption behavior of silver and palladium onto the extraction chromatographic LN resin was assessed by determining the weight distribution ratios (*D*_w_) over a wide range of HCl concentrations. To this purpose, the LN resin (50 mg) was weighted into 2 mL centrifugation tubes, and different concentrations of HCl solutions were added to each tube. The tubes were closed and shaken for 2 h to pre-condition the resin and an aqueous solution containing Ag(I) (10 µg, 100 µL) and Pd(II) (10 µg, 100 µL) was added (A_0_ sample). The final volume was 1 mL while the final HCl concentrations varied from 0.001 to 1 M. Each mixture was allowed to equilibrate for 24 h on an orbital shaker (Suprema Continental Instruments) at room temperature. The liquid was then withdrawn from each tube and filtered with a 0.2 μm pore RC membrane syringe filter to isolate the resin residue from the aqueous phase (A sample). Each aliquot was diluted appropriately and analysed by ICP-OES. Amount of Ag and Pd possibly retained by the membrane syringe filters was also assessed by ICP-OES by filtering solutions that have not been in contact with the resin and was subtracted to the computation of the aliquots’ concentration.

*D*_w_ values were calculated according to the following equation:

1 $$D_{{\text{w}}} ~ = \frac{{(C_{{{\text{A}}_{0} }} ~ \cdot ~V_{{{\text{A}}_{0} }} ) - (C_{{\text{A}}} \cdot V_{{\text{A}}} )~}}{{m_{{\text{R}}} }}$$

where ${C_{{{\text{A}}_{0} }} }$ is the metal (silver or palladium) concentration in the A_0_ sample, C_A_ is the metal concentration in the *A* sample, ${V_{{{\text{A}}_{0} }} }$ is the volume of the A_0_ sample, V_A_ is the volume of the A sample and *m*_R_ is the mass of the resin. Each *D*_w_ measurement was performed at least in triplicate.

### Silver-palladium chemical separation

The process of separating silver-111 from palladium target was developed through bench experiments using the two stable elements. The whole production procedure, including the dissolution of the target, was mimicked in order to obtain the conditions most similar to a real silver-111 production. The chromatographic separation was performed using LN resin (800 mg) packed on a Poly-Prep® chromatography column (Biorad, 9 × 4 × 0.8 cm) and a TK200 resin cartridge (2 mL). The resins were conditioned with 0.005 M HCl (50 mL) and 1 M HCl (5 mL), respectively.

A metallic Pd foil (30 mg) was dissolved in *aqua regia* (4 mL) while heating on a hot plate. The resulting solution was then evaporated to dryness, dissolved in HCl 1 M (3 mL) and evaporated again. This process was repeated 4 times to eliminate any trace of HNO_3_. The residue was then dissolved in a 0.005 M HCl (4 mL) solution containing 10 mg NaCl. The mixture was spiked with Ag(I) (100 µg, standard solution 1000 ± 2 mg/L) to simulate the dissolved irradiated material and loaded into the preconditioned LN column. The effluent was collected in the first fraction and a washing step with 0.005 M HCl (50 mL) was performed to ensure the removal of all Pd(II) from the resin. A 1 M HCl (20 mL) solution was then added to elute Ag(I). The separations were conducted using a semi-automated system with an ISMATEC peristaltic pump with a flow rate of 1 mL/min collecting fractions of around 10 mL. Fractions containing Ag(I) eluted from the LN resin column (around 20 mL) were loaded onto a preconditioned TK200 resin cartridge. The cartridge was rinsed with 1 M HCl (10 mL) and then Ag was eluted with pure water (5 mL). Aliquots of all the fractions collected during the separation were properly diluted and analyzed by ICP-OES. Each separation was repeated at least in triplicate. The optimized separation parameters were then applied for the purification of reactor-produced silver-111 batches.

### Silver-111 production

Silver-111 was produced *via* neutron irradiation of natural palladium targets at TRIGA Mark II nuclear research reactor at the Laboratory of Applied Nuclear Energy (LENA) of the University of Pavia (Italy), as recently reported by Morselli et al. (Morselli et al. 2023). Briefly, natural palladium targets (50 mg) were irradiated in the central thimble (CT) for 2 h with thermal neutron flux of 6.7·10^12^ cm^–2^ s^–1^ and total neutron flux in the CT of 1.7·10^13^ cm^–2^ s^–1^ at full reactor power (250 kW), producing 0.6 MBq of silver-111 and 580 MBq of palladium-109 at the end of the irradiation. Silver-111 was obtained via the ^110^Pd(n, γ)^111 m/g^Pd capture reaction and the subsequent beta-decay of palladium-111; palladium-109 was produced via the ^108^Pd(n, γ)^109 m/g^Pd capture reaction. The thermal activation cross sections of the reactions are about 3.8 b and 148.9 b, respectively (Krane 2019). The use of a natural palladium matrix was not considered detrimental for these tests because the formation of palladium-109 allowed the direct evaluation of the Ag/Pd separation by means of γ-spectrometry measurements. Short irradiation times were chosen in order to produce sufficient radioactivity to perform the radiochemical separations while sparing the exposure of the operators. It is worth to clarify that the TRIGA mark II nuclear reactor is a small reactor dedicated to research projects and it was employed herein to provide the amounts of silver-111 necessary to refine an Ag/Pd separation method devised with not radioactive materials. Although longest irradiations are feasible and higher amount of enriched palladium target could be employed, it is unlikely that the reactor will be used for a remarkable scale up of silver-111 production.

### Silver-palladium radiochemical separation

**Fig. 2:**
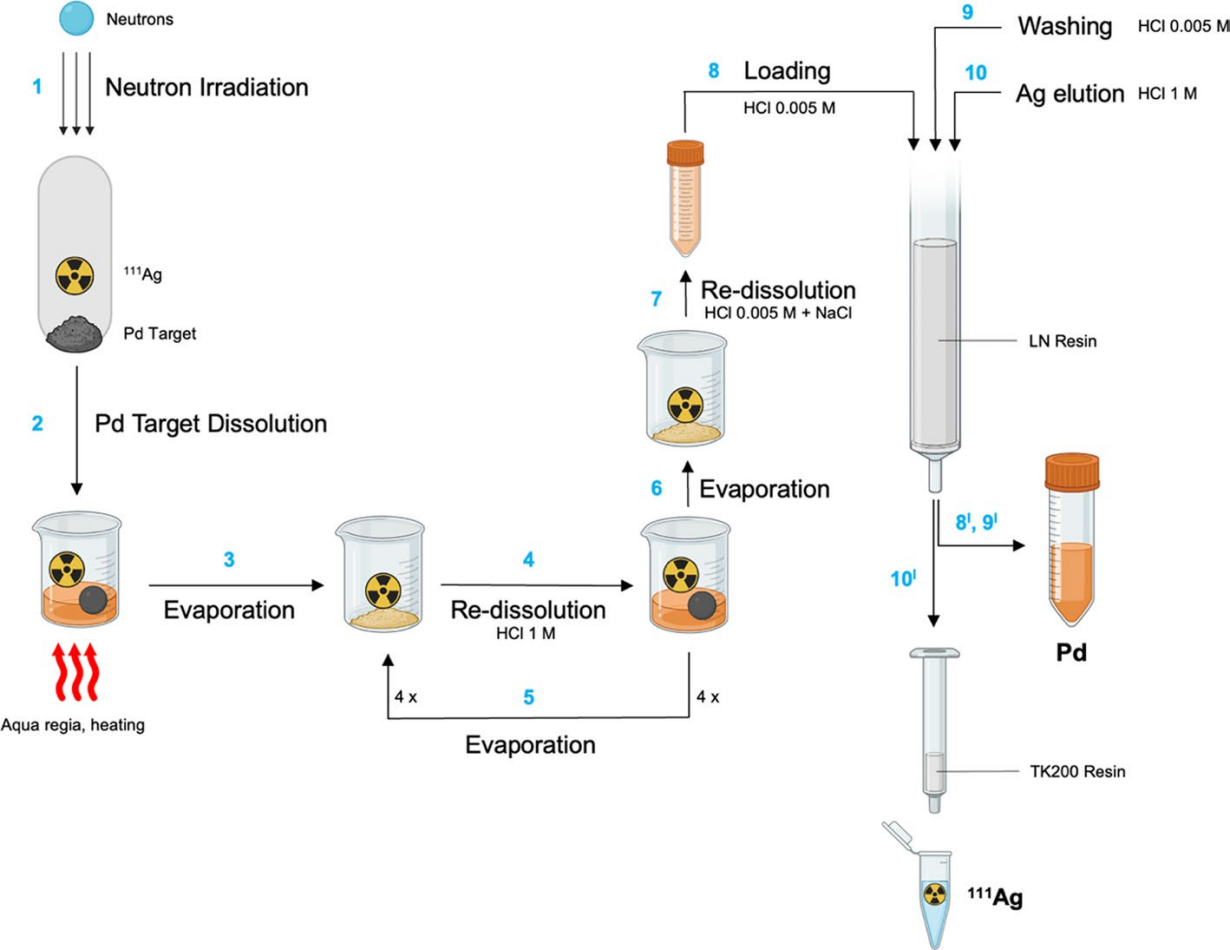
Schematic representation of the chromatographic separation process of silver-111 from palladium target developed in the present work. Image created with BioRender.com

The irradiated palladium target was dissolved using the same procedure described above and the radiochemical separation was conducted as previously described with non-irradiated materials with the only exception of the pump flow rate that was kept at 0.5 mL/min. Each radiochemical separation was monitored *via* γ-ray spectrometry by withdrawing an aliquot of each fraction, weighting it, and computing the silver-111 and palladium-109 contents by using the characteristic γ-lines in the spectra of the various isotopes. Dead time was kept below 5%. Spectra were analyzed using a GammaVision software. A schematic representation of the whole separation process is depicted in Fig. 2.

### Palladium target recovery

The target material separated from silver-111 was recovered after each purification process and recycled according to the procedure described by Ohya et al. (2020). Briefly, the palladium-containing fractions were evaporated to dryness and dissolved in a 0.1 M HCl solution (10 mL). The Pd(II) reduction to Pd(0) was conducted by adding NaBH_4_ (10 mL, 20%, 1 h) at ambient temperature. After centrifugation (1500 rpm, 5 min), the supernatant was discarded and washed with ultrapure water (20 mL). These processes were repeated four times, then ethanol (20 mL) and finally diethyl ether (20 mL) were used for the last rinsing. The precipitate was dried and quantitatively recovered.

## Results

### Silver-palladium weight distribution ratios

**Fig. 3:**
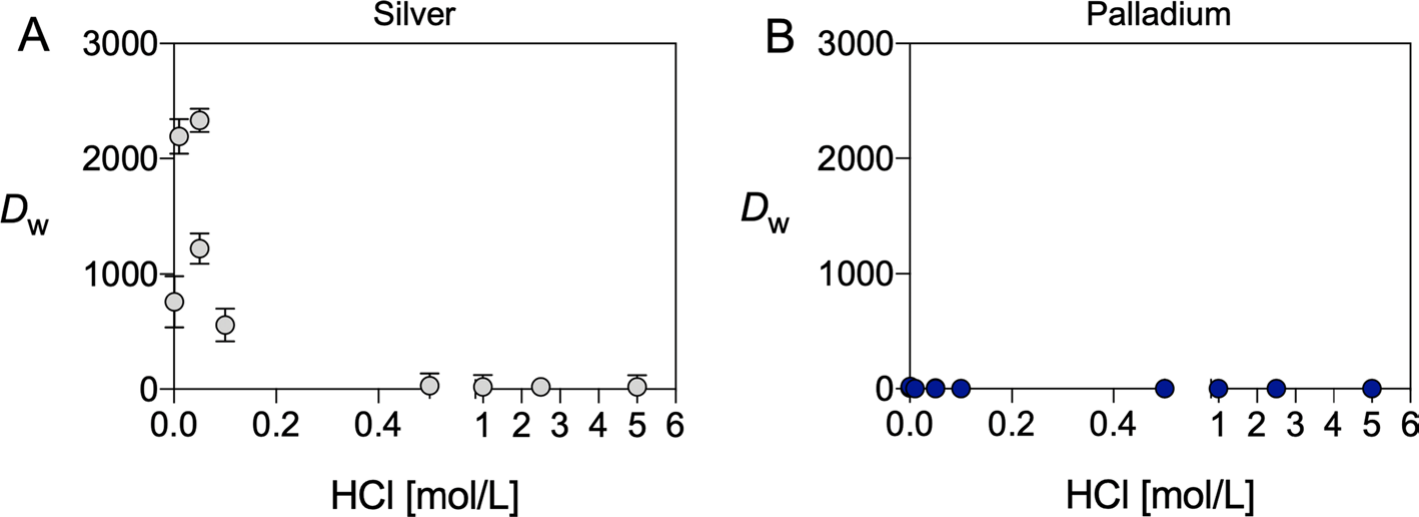
Weight distribution ratios (*D*_w_) for (**A**) Ag(I) and (**B**) Pd(II) in HCl solutions at several concentrations on LN extraction chromatography resin

As reported in Fig. 3A, the use of LN resin allowed to determine weight distribution ratios (*D*_w_) higher than 1000 for Ag(I) at HCl concentrations lower than 0.05 M whilst, under increased HCl molarity (> 0.2 M), *D*_w_ dropped notably to < 10. This indicates that Ag(I) could be strongly retained by the resin at highly dilute HCl conditions and that it could be subsequently stripped by increasing HCl concentration.

Conversely, as highlighted in Fig. 3B, minimal Pd uptake on LN resin was detected in any condition, with *D*_w_ values consistently below 10 across the entire concentration range.

### Silver-palladium chemical and radiochemical separation

**Fig. 4:**
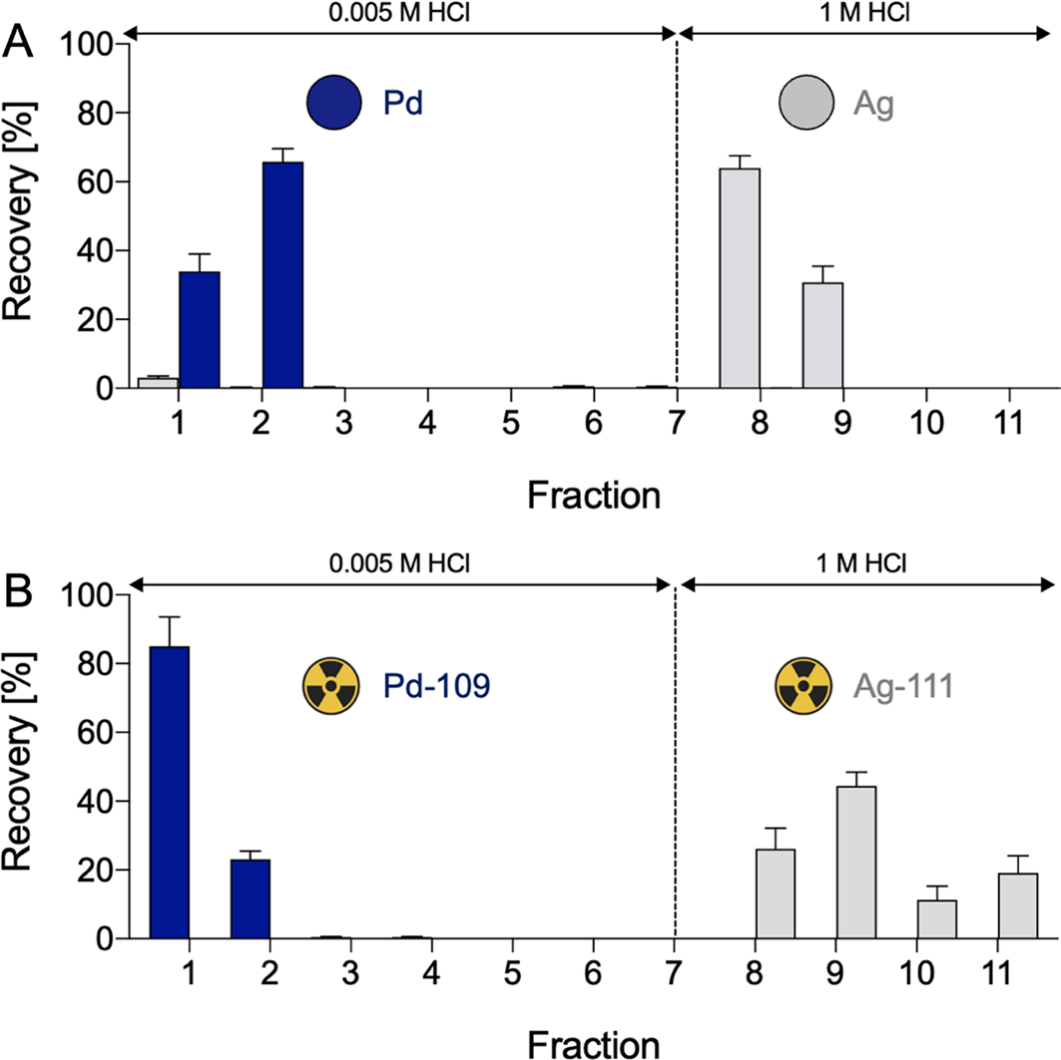
Elution profiles of silver and palladium on LN resin with (**A**) non-irradiated palladium target (metal amounts assessed by ICP-OES) and (**B**) irradiated palladium target (radiometal amounts assessed by γ-spectrometry). Fraction volume ~ 10 mL; fractions 1–7: 0.005 M HCl, fractions 8–11: 1 M HCl

The result of the Ag/Pd separation experiments using stable elements is reported in Fig. 4A. Following the process hereby described, Pd(II) was almost thoroughly removed in the loading step and during the first washing steps (25 mL, 0.005 M HCl) being not retained by the resin (Pd(II) recovery > 99.96%). The successive addition of 1 M HCl (40 mL) allowed to quantitatively retrieve Ag(I) in the first two fractions (20 mL, Ag(I) recovery > 95%). Silver purity assessed by ICP-OES measurement was > 99% with a separation factor (S_Pd,Ag_) of around 4.21·10^−4^.

**Fig. 5:**
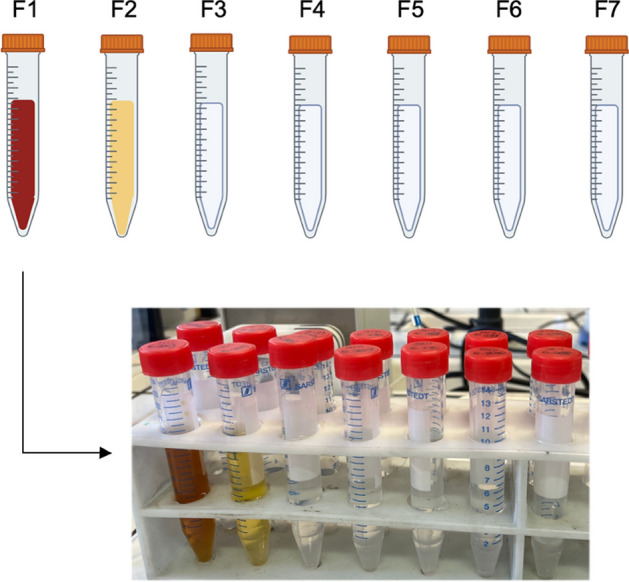
Visual demonstration of Pd(II)-Ag(I) separation

The progress of the separation could be also qualitatively evaluated by observing the color exchange of the eluted fractions: the Pd(II) chloro complexes have a marked reddish-brown color while the Ag(I) containing compounds are colorless in solution. A paradigmatic visual demonstration of the Pd(II) separation upon several eluent fractions is shown in Fig. 5.

The result of the silver-111/palladium-109 separation experiments by using irradiated palladium targets is reported in Fig. 4B. Similarly to the stable element experiment, palladium-109 was eluted in the first two fractions while silver-111 was stripped with around 40 mL of 1 M HCl. The recovery yields were determined by γ-spectrometry, directly tracking and quantifying the γ-emission of silver-111 and palladium-109 in every fraction and summing the activity attributed to the specific peaks after decay correction. Palladium-109 and silver-111 recovery was > 90% and > 92%, respectively. Radionuclidic purity, assessed by γ-spectrometry as well, was > 99%.

**Fig. 6:**
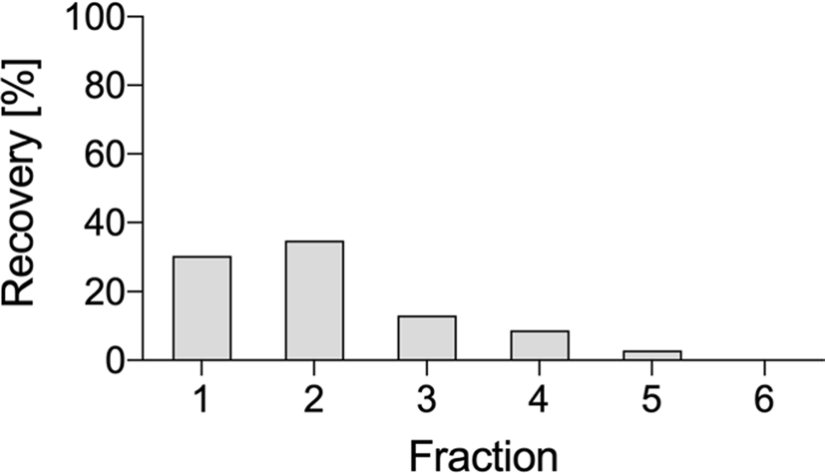
Elution profile of silver-111 on TK200 resin (fraction volume ~ 2 mL)

The loading of the silver-111 containing fractions onto a second column (TK200 resin cartridge) and following elution with water allowed both the concentration of the radiometal and a further purification step. As depicted in Fig. 6, around 95% of silver-111 activity was collected in 10 mL of eluent but ~ 80% was concentrated in the first three fractions (total volume = 6 mL).

## Discussion

This study presents the development of an innovative chemical separation method aimed at efficiently extracting silver-111 from bulk palladium targets.

In the initial phase, batch equilibrium experiments were conducted to explore the chemical behavior of Ag(I) and Pd(II) on the commercially available LN resin, and to identify the optimal conditions that would enable a highly efficient separation of the two metals. LN resin is made up of bis(2-ethylhexyl)phosphoric acid (HDEHP) compound, an extractant frequently used in the separation and recovery of lanthanides by solvent extraction and extraction chromatography (Ain et al. 2019; Monroy-Guzman et al. 2020; Zhang et al. 2022).

The obtained results demonstrated that, under the proper conditions, LN resin exhibited a marked Ag(I) selectivity over Pd(II). Specifically, at low HCl concentrations (from 0.01 to 0.05 M), Ag(I) was strongly adsorbed, leading to weight distribution ratios (*D*_w_) exceeding 1000. Conversely, under increased HCl molarity (> 0.2 M), Ag(I) showed undetectable retention. In sharp contrast, Pd(II) exhibited a negligible affinity for the LN resin at all HCl concentrations as the uptake remained minimal (*D*_w_ < 10) across the whole investigated range (Fig. 3). This distinct behavior has been ascribed to the different stability of the complexes formed with the stationary phase contained in the resin and the varying species formed by the two metals upon the whole range of HCl concentrations. According to the data presented in Table 1, at HCl concentrations below 0.1 M, Ag(I) largely exists in neutral ([AgCl]) and cationic (Ag^+^) forms that are able to strongly interact with the HDEHP groups and thus trigger the Ag(I) retention. Conversely, at the highest molarity, Ag(I) mainly exhibits a monoanionic chloro-complex form ([AgCl_2_]^−^) that showcases no retention of the extractant. A different scenario applies to Pd(II) where all the predominant chloro-complex species formed along the HCl concentration range (Table 1) are expected to be unretained by the resin, as they are negatively charged thus displaying a low ability to assemble stable interaction with the HDEHP groups. This hypothesis fails to interpret the behavior of Ag(I) in very low HCl concentrations (< 0.01 M) where, unexpectedly, the metals appears to display again a certain grade of releasing from the resin (Fig. 3). However, experimental errors cannot be totally excluded in this case as the *D*_w_ obtained does not afford a successful separation from Pd in an alternative set of experiments where Ag(I) was tentatively eluted from LN resin with pure water (data not shown).

**Table 1:** Percentages of Ag(I) and Pd(II) chloro-complexes computed to form at selected HCl concentrations (equilibrium calculations were performed based on the data reported in the IUPAC Stability Constant Database (“ScQuery” 2006)

HCl [mol/L]	Ag species			Pd species				
	Ag^+^	[AgCl]	[AgCl_2_]^−^	Pd^2+^	[PdCl]^+^	[PdCl_2_]	[PdCl_3_]^−^	[PdCl_4_]^2−^
0.005	6.4	63.9	29.7	0	3.8	58.1	36.6	1.5
0.1	0	9.7	90.2	0	0	4.2	53.3	42.5
1	0	1.1	98.9	0	0	0.1	11.1	88.8
6	0	0.2	99.8	0	0	0	2	98

Building upon these promising results, strongly suggesting that a separation of silver-111 from bulk palladium target could be devised *via* LN resin extraction chromatography, a column-based separation approach was established (Fig. 2).

The initial tests were firstly commenced with stable elements to test this innovative strategy. Natural metallic palladium foils were dissolved in *aqua regia* to simulate the Pd-target dissolution and then spiked with natural Ag(I) to mimic the small amount of silver-111 produced post-irradiation. The obtained solution was subsequently evaporated to dryness, with a follow-up repetition of the process using concentrated HCl to remove any trace of HNO_3_. The residue was then dissolved in a solution consisting of 0.005 M HCl co-added with NaCl. The addition of NaCl was necessary to achieve complete dissolution of the solid residue by the formation of anionic Ag(I) and Pd(II) chloro-complexes. This mixture was strategically chosen to elicit robust Ag(I)-complexes sorption onto the LN resin and, conversely, to let through Pd(II)-containing compounds. Ag(I)-containing species were then eluted using HCl concentrations > 0.5 M, as expected by the *D*_w_ values (*vide supra*). Dynamic separation experiments showed compelling evidence supporting the data deduced from the Ag/Pd equilibrium distribution coefficients: Pd(II) was efficiently removed (> 99%) using 0.005 M HCl in the loading and the first washing steps, while the successive additions of 1 M HCl allowed to efficaciously recover Ag(I) (> 95%) (Fig. 3A) providing a separation factor (S_Pd,Ag_) of around 4.21·10^−4^.

The LN resin-based chemical separation process was subsequently validated using a comparable amount of neutron-irradiated natural palladium targets (30 mg vs. 50 mg, respectively). After irradiation, the palladium matrix containing the mixture of co-produced silver-111 and palladium-109 was dissolved in *aqua regia* and processed using the same protocol described with stable metals. Then the solution was loaded into the LN column for the separation. It is important to mention that the irradiations were conducted in order to produce the minimal amount of radioactivity needed for this pivotal study. Increased irradiation times, sample mass, and the use of highly enriched palladium-110 targets lead to higher activity batches mainly carrying only Ag-111 (Morselli et al. 2023). These kinds of productions may be dedicated to future preclinical tests but the separation method described herein should not undergo significant variation.

**Fig. 7:**
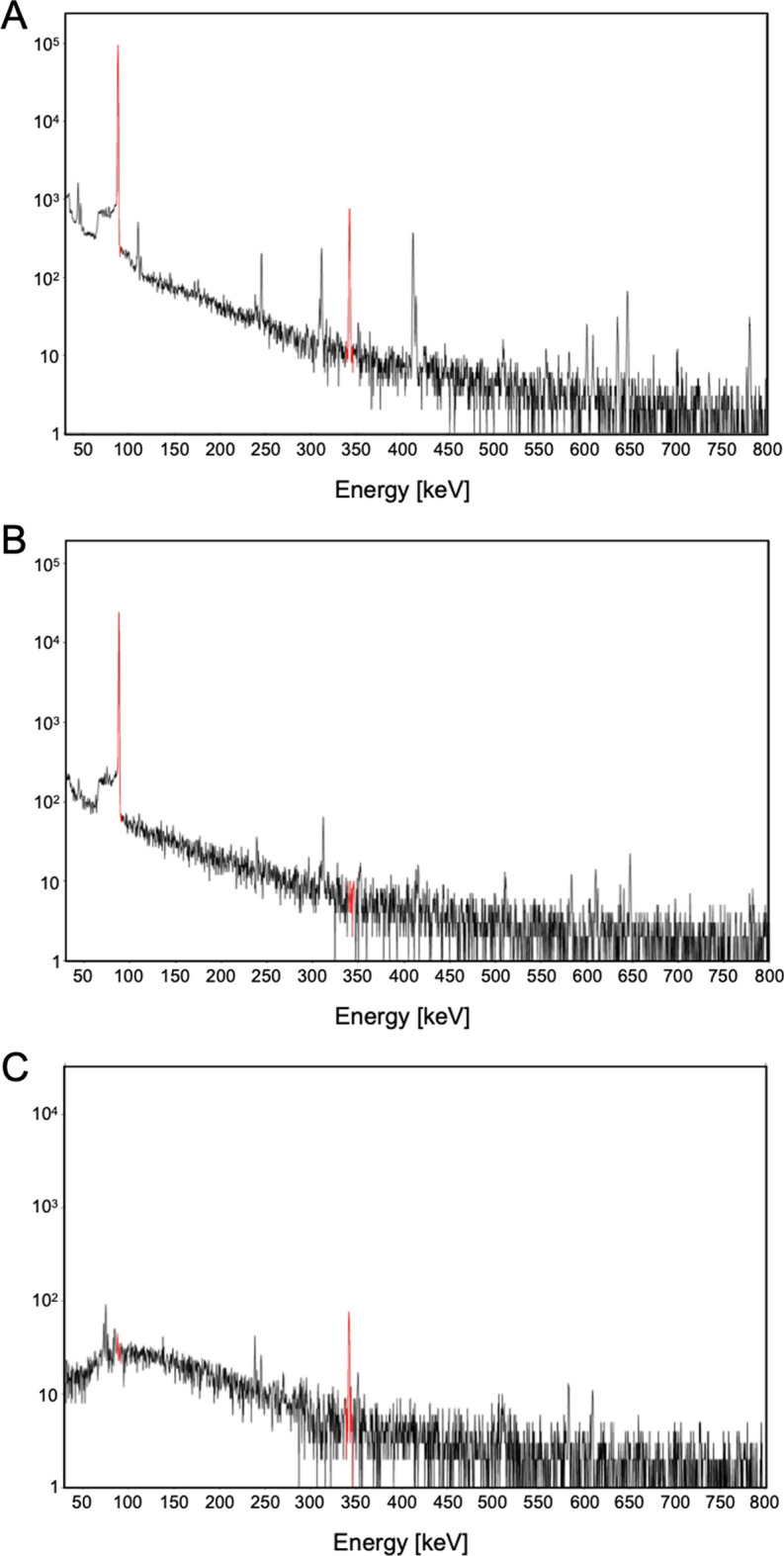
Paradigmatic γ-spectra of irradiated palladium target before the separation (**A**) and after the separation of palladium-109 (**B**) and silver-111 (**C**) containing fractions. Characteristic γ-ray emissions of purified palladium-109 and silver-111 are highlighted as red peaks at 88 keV and 342.1 keV energy, respectively

As depicted in Fig. 3B, the elution profiles obtained using the irradiated material were very similar to those obtained using the stable elements. The only notable difference observed was the larger volume needed for the elution of silver-111, which was tentatively attributed to the lower pump flow rate (0.5 mL/min vs. 1.0 mL/min, respectively). However, this minor difference did not affect the overall effectiveness of the separation process. The average recovery yield, calculated on the initial silver-111 activity theoretically computed and measured after irradiation, was impressively up to 92%, confirming a highly efficient separation. In Fig. 7, the γ-spectra of the mixture obtained after target dissolution and before the separation (A) and the γ-spectra of the separated palladium-109 (B) and silver-111 (C) fractions are shown. Crucially, negligible presence of palladium-109 is noticeable in the recovered silver-111 fractions after purification, attesting a radionuclidic purity exceeding 99% and serving as a conclusive indicator of the method’s effectiveness.

Following the successful separation using LN resin, an additional step was implemented to further decrease the solution volume and provide silver-111 in a ready-to-use formulation for labelling purposes. In fact, large volumes of 1 M HCl solutions give rise to notable concerns in the [^111^Ag]-labelling of chelator tethered to bioconjugates, as a buffered pH is usually needed for achieving a quantitative yield (Tosato et al. 2020, 2023). TK200 resin, a substrate containing Tri-Octyl Phosphine Oxide (TOPO) as extractant, was hence chosen for this purpose as it is widely used in liquid-liquid extraction methods, especially in the extraction of actinides from acidic media (Wang et al. 2019). Moreover, this resin is already acquainted in the field of radiochemistry as it is employed, together with ZR resin or TK400 resin, to separate gallium-67/68 from zinc targets and in the separation of zinc isotopes from copper targets (Bombard et al. 2018; Rodnick et al. 2020). The documented ability to interact with Cu(II) was particularly interesting for the application herein addressed since Cu(II) is one of the main contaminants of palladium targets (around 10 ppm and 770 ppm for natural palladium and palladium-110 enriched targets, respectively, based on the palladium data sheets). Actually, preliminary tests showed promising selectivity for Ag(I) and Cu(II) in TK200 resin hinting at different retention under HCl solutions effluents (data not shown).

Silver-111 containing fractions were loaded into the cartridge and subsequently stripped with pure water. Although the complete elution of Ag(I) requested around 10 mL of eluent, the harvesting of the first 6 mL provided a formulation suitable for direct radiolabeling of bioconjugates still ensuring an average yield of 80 ± 5%. The molar activity of the obtained silver-111 was estimated not less than 20 KBq/nmol and was reckoned considering the amount of silver contained as an impurity in the palladium target (50 ppm based on the palladium data sheets) and the silver possibly introduced during the separation as the only source of stable silver in the whole process. It is worth mentioning that this evaluation is only indicative and outcomes that are more reliable could be achieved only after punctual quantification of the stable silver amount by ICP-MS or ICP-OES analysis. Moreover, the molar activity herein computed is not representative of a real silver-111 production since the use of highly enriched palladium-110 targets (silver contents < 1 ppm), increased irradiation times, and implants with higher neutron flux, would indisputably lead to a higher production and hence to a much higher molar activity upon the separation conditions described.

## Conclusion

In this work, a new method to separate silver-111 from neutron irradiated palladium target was successfully devised using a two-step extraction chromatography. The obtained results demonstrated that silver-111 can be recovered with high yield and purity from the bulk palladium matrix. Our newly developed method represents a significant improvement over the previously reported procedures as the final recovery of silver-111 in a small volume of water enables its immediate use in the labeling of sensitive vectors without the need for time-consuming evaporation and reformulation steps. This advancement not only enhances efficiency but also significantly reduces the risk of introducing environmental contamination, ensuring the high purity of the final product. These findings open new avenues for advancing the utilization of silver-111 labelled radiotracers in therapeutic applications, so driving progress in cutting-edge scientific research with this fascinating radiometal.

## Data Availability

The data supporting the conclusions of this article are included in the manuscript.

## Abbreviations

HDEHP: Bis(2-ethylhexyl)phosphoric acid
ICP-OES: Inductively coupled plasma optical emission spectroscopy
ISOL: Isotope separation on-line
ISOLPHARM: ISOL technique for radioPHARMaceuticals
PET: Positron emission tomography
SPECT: Single photon emission computed tomography
TOPO: Tri-Octyl phosphine oxide
